# Multi-omics machine learning to study host-microbiome interactions in early-onset colorectal cancer

**DOI:** 10.1038/s41698-024-00647-1

**Published:** 2024-07-17

**Authors:** Thejus T. Jayakrishnan, Naseer Sangwan, Shimoli V. Barot, Nicole Farha, Arshiya Mariam, Shao Xiang, Federico Aucejo, Madison Conces, Kanika G. Nair, Smitha S. Krishnamurthi, Stephanie L. Schmit, David Liska, Daniel M. Rotroff, Alok A. Khorana, Suneel D. Kamath

**Affiliations:** 1 Department of Hematology-Oncology, Taussig Cancer Institute, Cleveland Clinic, Cleveland, OH USA; 2 Department of Medical Oncology, Dana-Farber Cancer Institute, Boston, MA USA; 3 Microbial Sequencing & Analytics Resource (MSAAR), Lerner Research Institute, Cleveland Clinic, Cleveland, OH USA; 4 Department of Quantitative Health Sciences, Lerner Research Institute, Cleveland Clinic, Cleveland, OH USA; 5 Center for Quantitative Metabolic Research, Cleveland Clinic, Cleveland, OH USA; 6 Department of Surgery, Cleveland Clinic, Cleveland, OH USA; 7 Case Comprehensive Cancer Center, Cleveland, OH USA; 8 Department of Hematology-Oncology, University Hospital Seidman Cancer Center, Cleveland, OH USA; 9 Center for Young-Onset Colorectal Cancer, Cleveland Clinic, Cleveland, OH USA; 10 Genomic Medicine Institute, Lerner Research Institute, Cleveland Clinic, Cleveland, OH USA; 11 Population and Cancer Prevention Program, Case Comprehensive Cancer Center, Cleveland, OH USA; 12 Department of Colorectal Surgery, Digestive Disease & Surgery Institute, Cleveland Clinic, Cleveland, OH USA

**Keywords:** Biomarkers, Translational research, Colorectal cancer, Metabolomics, Sequencing

## Abstract

The incidence of early-onset colorectal cancer (eoCRC) is rising, and its pathogenesis is not completely understood. We hypothesized that machine learning utilizing paired tissue microbiome and plasma metabolome features could uncover distinct host-microbiome associations between eoCRC and average-onset CRC (aoCRC). Individuals with stages I–IV CRC (*n* = 64) were categorized as eoCRC (age ≤ 50, *n* = 20) or aoCRC (age ≥ 60, *n* = 44). Untargeted plasma metabolomics and 16S rRNA amplicon sequencing (microbiome analysis) of tumor tissue were performed. We fit DIABLO (Data Integration Analysis for Biomarker Discovery using Latent variable approaches for Omics studies) to construct a supervised machine-learning classifier using paired multi-omics (microbiome and metabolomics) data and identify associations unique to eoCRC. A differential association network analysis was also performed. Distinct clustering patterns emerged in multi-omic dimension reduction analysis. The metabolomics classifier achieved an AUC of 0.98, compared to AUC 0.61 for microbiome-based classifier. Circular correlation technique highlighted several key associations. Metabolites glycerol and pseudouridine (higher abundance in individuals with aoCRC) had negative correlations with *Parasutterella*, and *Ruminococcaceae* (higher abundance in individuals with eoCRC). Cholesterol and xylitol correlated negatively with *Erysipelatoclostridium* and *Eubacterium*, and showed a positive correlation with *Acidovorax* with higher abundance in individuals with eoCRC. Network analysis revealed different clustering patterns and associations for several metabolites e.g.: urea cycle metabolites and microbes such as *Akkermansia*. We show that multi-omics analysis can be utilized to study host-microbiome correlations in eoCRC and demonstrates promising biomarker potential of a metabolomics classifier. The distinct host-microbiome correlations for urea cycle in eoCRC may offer opportunities for therapeutic interventions.

## Introduction

The rates of early-onset colorectal cancer (eoCRC) have increased in recent years, but the etiology and pathogenesis are not well understood^1–5^. In the United States, eoCRC disproportionately affects racial and ethnic minorities, has a greater chance of being detected at advanced stages, and presents with challenges that are unique to younger people (fertility preservation, impact on job security, and lifelong effects on quality of life due to treatment toxicities)^3,6^. While there is certainly a benefit in timely diagnosis and treatment, universal population-level screening with conventional methods may not be a cost-effective solution to address the rising incidence of eoCRC^7^. Therefore, there is a need to better understand the pathogenesis of eoCRC and identify biomarkers for risk-adaptive screening for cancer prevention as well as for cancer directed therapy^8^.

Recent advances in understanding the microbiome and metabolome hold promise in this direction. It is increasingly recognized that exposures to environmental factors, lifestyle, and diet alter our bodies’ microbiome^2^. Metabolomics identifies alterations in chemical reactions at the cellular level resulting from these exposures, changes related to pathologic processes such as carcinogenesis, and those resulting from the microbiome^9^. These alterations may serve as markers for cancer development and thus be utilized to identify individuals at increased risk for developing cancer^10^. We and others have previously demonstrated the significance of microbiome and metabolome in carcinogenesis and have shown unique associations of these features with eoCRC^11,12^. By using advanced multi-omic analytic techniques, new insights into their relationships can be gained. Our study aims to study the feasibility of using machine learning classifiers based on microbiome and metabolome features as well as network analysis to uncover distinct host-microbiome associations related to eoCRC compared to aoCRC.

## Results

### Baseline characteristics

The study population comprised of 64 individuals with CRC (*N* = 20 eoCRC and *N* = 44 aoCRC). The majority were male—60% for eoCRC vs. 59% aoCRC, and White—100% vs. 91%. Tumors were mostly left-sided (85% eoCRC vs. 68% aoCRC) and rectal primary (65% vs. 39%). Stage IV disease comprised 45% of those with eoCRC vs. 20% of aoCRC. Differences in baseline characteristics were not statistically significant (*p* > 0.05 for all).

Most non-cancer controls were individuals with liver adenomas (*n* = 15, 31.3%), healthy donors (*n* = 11, 22.9%), or those with other benign liver conditions such as cysts (*n* = 10, 20.8%), hemangiomas (*n* = 7, 14.6%) and focal nodular hyperplasia (*n* = 5, 10.4%). The baseline characteristics for both groups are outlined in Table 1.

**Table 1:** Baseline characteristics of population selected for the analysis—*n* (%) for categorical variables and median (interquartile range—IQR) for continuous variables^a^

Characteristics	eoCRC (*n* = 20)	aoCRC (*n* = 44)	Control (*n* = 49)
Sex			
Male	12 (60%)	26 (59%)	8 (16.3%)
Female	8 (40%)	18 (41%)	41 (83.7%)
Comorbidities			
Hyperlipidemia	5 (25%)	12 (27%)	26 (53.1%)
Obesity	5 (25%)	3 (7%)	25 (51.0%)
Diabetes	1 (5%)	13 (30%)	5 (10.2%)
Tumor characteristics			
Left sided	17 (85%)	30 (68%)	–
Stage IV disease	9 (45%)	9 (20%)	–

*CRC* colorectal cancer, *eoCRC* early-onset colorectal cancer, *aoCRC* average-onset colorectal cancer.

^a^*p* > 0.05 for all comparisons.

### Discriminative clustering of metabolomics and microbiome features in multiomics models

**Fig. 1:**
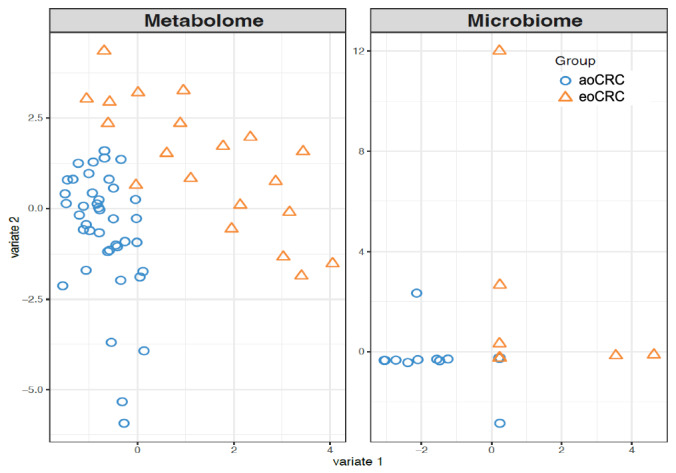
DIABLO dimension reduction demonstrating discriminative clustering of metabolomics and microbiome features in eoCRC vs. aoCRC.

Distinct clustering patterns were observed in the multi-omic dimension reduction plots as outlined in Fig. 1. The area under the curve (AUC) plots (Supplementary Fig. 1a, b) confirmed the superior performance of the metabolomics classifier (AUC 0.98) vs. the microbiome classifier (AUC 0.61). Combining the microbiome and metabolome data resulted in an intermediate AUC of 0.83.

### Metabolomic and microbiome features involved in eoCRC molecular profile

**Fig. 2:**
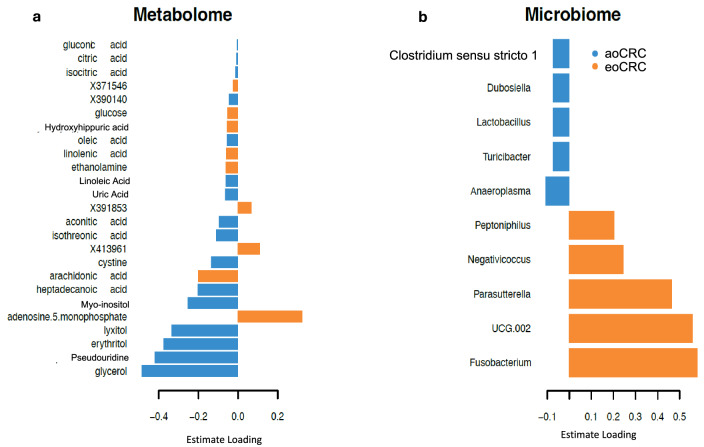
Discrimination of cancer groups using PLS-DA. Loading plot from the PLS-DA applied to the (**a**) Metabolome and (**b**) Microbiome data to discriminate cancer groups. Colors indicate the group in which the median is maximum for each feature.

Block Rank algorithm-based ranking revealed several unique metabolites and microbiome features contributing to the model (Fig. 2). The features and the estimate loading are represented in Fig. 2. These included distinctive metabolites (*n* = 25, e.g., glycerol, pseudouridine, adenosine-5-monophosphate) and microbial taxa (*n* = 10, e.g., *Fusobacterium, Ruminococcaceae UCG 002, Parasutterella, Anaeroplasma*).

### Metabolomic-microbiome interactions unique to eoCRC

**Fig. 3:**
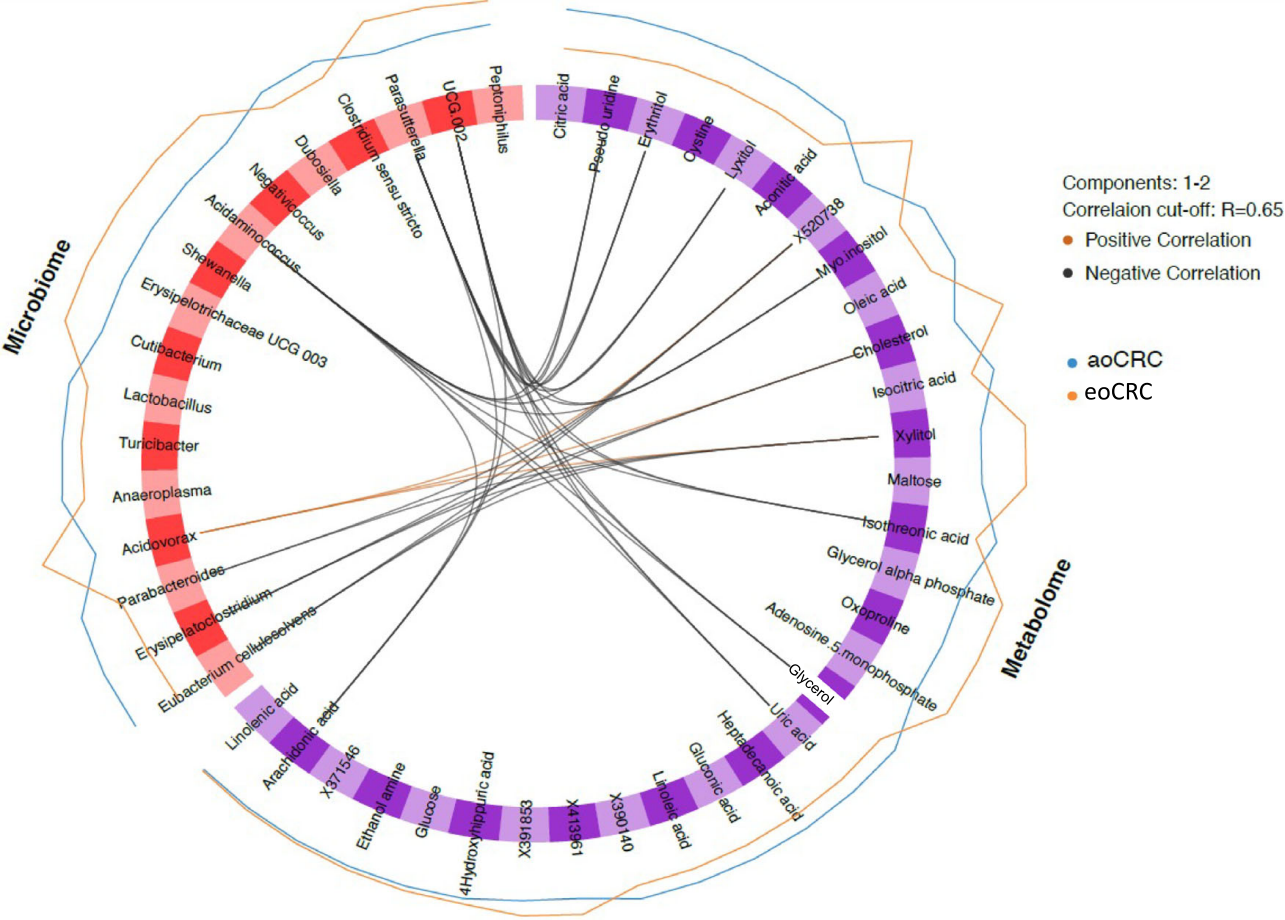
Circular correlation analysis of key metabolite-microbial genera associations (positive in red, negative in black).

Circular correlation technique analysis (Fig. 3) highlighted several key associations between metabolites and microbial genera in eoCRC vs aoCRC. For example, metabolites glycerol and pseudouridine (greater abundance in aoCRC) negatively correlated with microbial taxa *Parasutterella* and *Ruminococcaceae UCG 002* (lower abundance in aoCRC). Both of these metabolites also negatively correlated with *Acidaminococcus*. Other metabolites negatively correlated with these microbiome taxa (*Parasutterella, Ruminococcaceae UCG002 and Acidaminococcus*) were erythritol, lyxitol, myoinositol, uric acid, and arachidonic acid. *Parabacteroides, Erysipelatoclostridium*, and *Eubacterium cellulosolvens* negatively correlated with xylitol while the latter two (*Erysipelatoclostridium* and *Eubacterium*) also negatively correlated with cholesterol. Positive correlations were identified for *Acidovorax* (more abundant in eoCRC) with cholesterol and xylitol. Several metabolites found relevant in the block rank algorithm such as adenosine-5-monophosphate and hydroxyhippuric acid were not correlated with the microbiome.

### Comparison with controls

Only plasma metabolomics was performed for controls. The metabolomics-based classifier for the non-CRC controls had a much lower AUC of 0.78 (in making young vs. old classification) as opposed to the AUC of 0.98 for people with CRC. In assessing the correlation of metabolites with age, no significant associations were found (Supplementary Fig. 2).

### Post hoc analysis using selected metabolites and microbiome

Tukey’s Honest Significant Difference test (post hoc) using selected metabolites and microbiome was performed to assess metabolites of relevance in the machine learning model and showed that the age-related variation is not significant (*p* value = 0.369).

### Network analysis

**Fig. 4:**
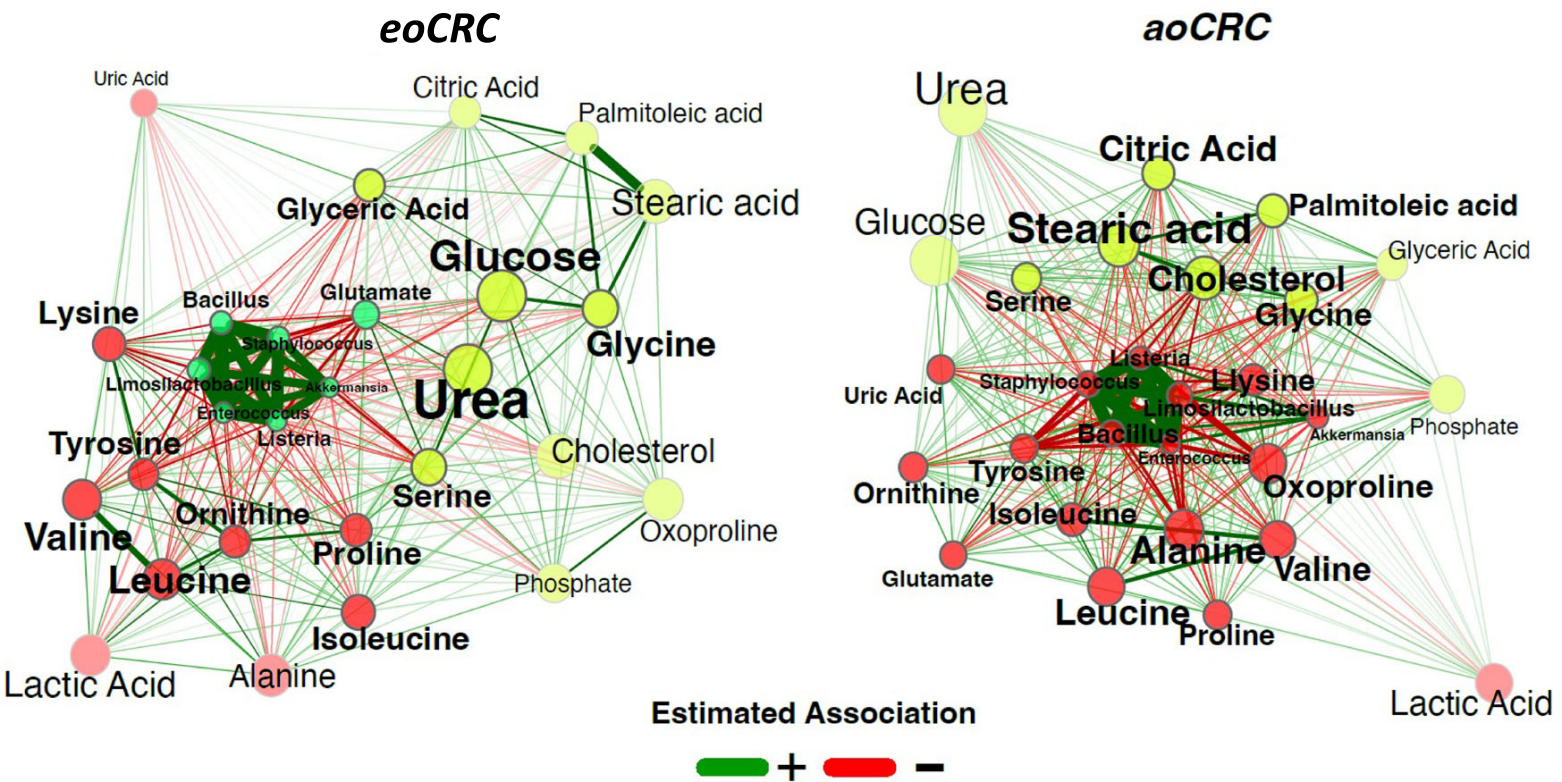
Network analysis of metabolomic–microbiome correlations of eoCRC vs. aoCRC. The SPRING method was used as an association measure. The estimated partial correlations are transformed into dissimilarities via the “signed” distance metric and the corresponding similarities are used as edge weights. Eigenvector centrality is used for defining hubs and scaling node sizes. Node colors represent clusters, which are determined using greedy modularity optimization. Clusters have the same color in both networks if they share at least two taxa. Green edges correspond to positive estimated associations and red edges to negative ones. Nodes that are unconnected in both groups are removed.

In our network analysis, we applied a comprehensive approach by integrating microbial taxonomy and metabolomics features, avoiding preselection biases from the DIABLO model. The method revealed distinctive clustering patterns which are represented in Fig. 4.

Briefly, the SPRING method was used as an association measure, transforming estimated partial correlations into dissimilarities and using the corresponding similarities as edge weights^13^. Eigenvector centrality defined hubs, the scaled node sizes, and corresponding label sizes, while node colors represented clusters determined using greedy modularity optimization. Green edges denoted positive estimated associations and red edges to negative ones. Unconnected nodes in both groups were removed.

Metabolites of the urea cycle, including urea and uric acid, showed different clustering patterns and centrality in the two groups. Notably, urea and ornithine had higher centrality in eoCRC versus aoCRC. Citric acid exhibited greater centrality in aoCRC compared to eoCRC, while its related metabolite glutamate showed different clustering patterns between the groups. The centrality of the microbe *Akkermansia* differed in the two groups, showing stronger negative associations with metabolites serine and glutamate in eoCRC compared to aoCRC.

## Discussion

We used machine learning classifier models to characterize the multi-omics features of eoCRC vs. aoCRC and found that plasma metabolomic features separated the cancer cohorts by age of onset more efficiently than tumor microbiome features. The metabolomics-based machine learning model had better discriminatory power, or the ability to effectively classify eoCRC vs. aoCRC using features from the training dataset. The lack of similarly efficient classification in the non-CRC control group suggests that the alterations associated with CRC contributed to this distinction. Further, using a multi-omics approach, we demonstrated that several metabolites of relevance in CRC and the classifier models exhibited correlations with multiple microbial taxa, highlighting differences between eoCRC and aoCRC. These include pseudouridine, glycerol, cholesterol, myoinositol, and arachidonic acid^14–20^. Therefore, it appears that the alterations in plasma metabolome are partly explained by differences in the tumor-associated microbiome, which may also explain the variations in metabolites observed between studies^21^. The fact that the microbiome only partially accounts for the eoCRC vs aoCRC differences is also reflected in the lower AUC for the microbiome-based classifier. In the present study, differential correlations in metabolites such as erythritol, which don’t have a well-established association with colorectal cancer, were identified and need further investigation^22^. Additionally, certain metabolic differences such as adenosine-5-monophosphate and 4-hydroxyhippuric acid that are of relevance in cancer were not correlated with the microbiome, suggesting host-driven metabolic influences for these metabolites^12,23,24^. Network analysis revealed a distinct clustering of urea cycle metabolites with the microbiome in eoCRC compared to aoCRC. This finding is interesting in light of the urea cycle’s association with CRC and its potential therapeutic relevance^25,26^.

Although metabolomics was able to capture the eoCRC vs aoCRC distinction more comprehensively, studying the differentially altered microbiomes is also relevant as the significance of microbiome alterations in the pathogenesis of eoCRC is increasingly understood. Factors such as antibiotics and dietary patterns are believed to contribute to dysbiosis, which can alter gene expression and the immune microenvironment, leading to cancer^27,28^. Dysbiosis is particularly relevant in eoCRC given the birth cohort effect (increasing incidence of eoCRC since the early 1950s), which may be related to environmental exposures that manifest in the microbiome^7^. Therefore, microbiome signatures are being sought as potential biomarkers with the potential to complement existing colorectal cancer screening tools^29^. The microbes thought to have carcinogenic potential in CRC include *Fusobacterium, Anaeroplasma, Flavonifractor, Parasutterella, Ruminococcaceae UCG 002, Acidovorax, Anaeroplasma, and Eubacterium*^9,30–38^. We therefore analyzed the microbiome features contributing to the classifier models. The microbial taxa significant in the correlative analysis were *Parasutterella* and *Ruminococcaceae UCG 002*, which were more abundant in eoCRC. Other microbes contributed to the model (*Fusobacterium, Anaeroplasma*) but did not meet the threshold for metabolic correlations, while several microbes correlated with metabolite differences but did not contribute to the model (*Acidaminococcus, Acidovorax, Erysipelatoclostridium, Eubacterium cellulosolvens*).

A prior study attempted a similar application of multi-omics utilizing fecal metabolomics and demonstrated the distinct metabolomic and microbiome features of eoCRC versus aoCRC and in comparison to subjects without a history of colon cancer^9^. The fecal metabolites that were relevant in the study included microbiota derivatives of tryptophan and bile acid metabolites^9^. While our findings differed from this prior study, possibly reflecting the variations in sampling, we demonstrated that a multi-omic approach incorporating plasma metabolomics and tissue microbiome is feasible. In a related investigation, a metabolomics-based classifier achieved an impressive area under the curve (AUC) of 0.81 for the diagnosis of colon cancer and 0.89 for rectal cancer, respectively^39^. Notably, diagnostic accuracy for cancer was substantially enhanced when metabolite markers were integrated with protein markers (specifically, CEA and CA19-9), resulting in an elevated AUC of 0.94 for both colon and rectal cancer^39^.

The finding from the network analysis pertaining to urea cycle and eoCRC is biologically relevant and worth investigating further. Association of elevated urea levels with lower levels of urea metabolizing microbe *Bifidobacterium*, and its contribution to colorectal carcinogenesis has been previously established^25^. The authors of the same study also demonstrated how pharmacologic inhibition of urea cycle metabolism and *Bifidobacterium longum* supplementation reduced murine intestinal tumor numbers and sizes. *Bifidobacterium* was not a key microbe in our model, likely due to different sampling (tissue microbiome in the present study vs fecal microbiome in the other). However, it appears that urea cycle is relevant especially in eoCRC. The metabolites involved in urea cycle are also part of Arginine biosynthesis pathway with demonstrated association with CRC including eoCRC and potential therapeutic relevance^26^. Whether the distinct clustering patterns observed in eoCRC vs aoCRC suggest distinct pathogenetic processes remains to be validated. The alterations of other metabolites such as citric acid and microbes such as *Akkermansia* in eo vs. aoCRC have been previously described^11,12^. In addition, the present study demonstrates how *akkermansia* may influence the differential expression of certain metabolites. The findings continue to indicate the underlying metabolic and microbiome differences that need further investigation.

Our study has several limitations. First, although our findings of metabolomics as a robust classifier are consistent with prior studies, we did lack an external cohort for validation. In the study we focused on demonstrating the feasibility of multi-omic analysis to uncover distinct host microbiome associations in eoCRC versus aoCRC. Since we are studying the feasibility of this approach and required multiple data points on the same group of individuals with CRC (metabolomics, tissue microbiome, clinical characteristics) which are not uniformly available across different datasets, we limited our study to a single institution. However, we included a control group of individuals without CRC to isolate the effect of CRC on the metabolome and demonstrated that the findings were unique to people with CRC. Second, correlative findings of metabolomics and microbiome were made, but it is unclear how much of the relationships observed are causal versus associative. Given the timing of the sample collection for the present study, the models describe molecular profiles after the disease is established rather than predicting its occurrence. While even establishing associations is relevant from a biomarker perspective, longitudinal analysis and mechanistic studies are crucial to elucidate the relationships from a pathogenesis standpoint and understand the biological implications^40,41^. Lastly, given the retrospective nature of the study, the impact of unmeasured confounders cannot be determined. However, we did evaluate potential confounders and demonstrated that they were not significantly different in the different groups that were compared. Third, the limitations of the models (overfitting of metabolomics model and low AUC for microbiome model) could influence the results. We did consider other classical machine learning approaches that are robust to overfitting and decided to use the DIABLO approach which has an established role in multiomics analysis^42,43^. Additionally, network analysis was performed independent of the classifier models and demonstrated unique metabolomic-microbiome correlations.

The current study presents several notable findings of future relevance. First, the metabolomics classifier exhibited remarkable performance, demonstrating its potential as a robust biomarker for precise risk assessment and the development of effective therapeutic interventions. Second, our findings underscore the potential of metabolomics for enhancing the assessment of pathophysiological alterations and providing a comprehensive perspective by incorporating facets of host metabolism and dysbiosis. Third, our multi-omics analysis unveiled pronounced differences in host-microbiome interactions between eoCRC and aoCRC, providing insights into the pathogenic mechanisms that could be investigated further. This is relevant given the increasingly recognized role of dysbiosis in carcinogenesis and aligns with ongoing efforts to utilize microbiome modulation as a viable approach in cancer therapy^44–46^. Modulation of urea cycle-related microbes to influence the natural history of colorectal cancer is one such area of interest and our findings concerning age-specific differences add another dimension that needs investigating^25^.

In conclusion, our findings demonstrate that multi-omics analysis can help identify unique host-microbiome interactions associated with eoCRC and aoCRC. The strategy can help identify biomarkers for screening and treating eoCRC.

## Methods

### Samples

Plasma samples were obtained from the prospective colorectal and liver tumor biobanks at Cleveland Clinic from 01/2004 to 03/2021. The liver biobank was included because it was the source of non-CRC control blood samples. Samples were obtained from individuals on the day of their procedures, inventoried, and immediately stored at −80 °C. The samples were maintained at −80 °C until processing. The study was performed under the oversight of the Cleveland Clinic institutional review board and the ethical approval process (IRB # 4134 and IRB#10-347), and written informed consent was obtained from all human participants. This study was conducted in accordance with the guidelines of the Declaration of Helsinki, the Belmont report, and the U.S. Common rule.

The samples included cases (individuals with a diagnosis of CRC) and controls (individuals without CRC or any other malignancy). The cases comprised all stages of CRC. Case samples were obtained at the time of surgical resection of the primary disease. Control samples included those who underwent liver resections or biopsies for benign causes, or liver transplant donors. The cohort was categorized based on the ages at the time of diagnosis as age ≤ 50 years (individuals with eoCRC or young non-CRC controls) or age ≥ 60 years (individuals with aoCRC or older non-CRC controls). Clinical information was obtained from a review of electronic medical records. Of note the control samples were used only for metabolomic analyses.

### Metabolomic analysis

The samples underwent metabolomic analyses using gas chromatography time-of-flight mass spectrometry (GC-TOF-MS) with the Primary Metabolism panel from West Coast Metabolomics at University of California, Davis^47^. This assay is designed for untargeted plasma analysis and detects over 200 known and more than 200 unknown metabolites. The list of known metabolites is accessible at https://metabolomics.ucdavis.edu/core-services/metabolites^48^. Previous publications have provided comprehensive information on the technique’s validity and procedures related to plasma extraction and metabolomics^49–52^.

Briefly, the samples were subjected to extraction using a solution consisting of acetonitrile, isopropanol, and water in a ratio of 3:3:2, which was chilled to −20 °C and degassed. A volume of 1 ml of this solution was utilized for extraction. For metabolite derivatization, a two-step process previously described was employed^49^. First, methoximation was used to protect carbonyl groups, followed by the exchange of acidic protons with trimethylsilyl groups to enhance volatility. A sample was then injected into an Agilent 6890 GC (Agilent Technologies, Santa Clara, CA, USA), equipped with a Restek Rtx-5Sil MS column (30 m × 0.25 mm, 0.25 μm) and operated with a splitless time of 25 s and a helium gas flow rate of 1 ml/min. The oven temperature was initially held at 50 °C for 1 min and then increased to 330 °C at a rate of 20 °C/min, where it was maintained for 5 min.

Data acquisition was carried out using a Leco Pegasus IV time-of-flight mass spectrometer (Leco Corporation, St. Joseph, MI) with electron ionization at −70 eV. Raw data were processed using ChromaTOF version 4.50, which included baseline subtraction, deconvolution, and peak detection. Metabolite annotation and reporting were performed using Binbase^53^.

### Microbiome analysis

16S rRNA gene amplicon sequencing and bioinformatics analysis were conducted following previously established protocols from colonic tissue specimens obtained at the time of surgical resection^11,54,55^. In brief, raw 16S amplicon sequences and metadata were demultiplexed using the split_libraries_fastq.py script within the QIIME2 software^56^. The demultiplexed fastq files were subsequently divided into sample-specific fastq files using the split_sequence_file_on_sample_ids.py script in QIIME2. Subsequently, individual fastq files were processed using the Divisive Amplicon Denoising Algorithm (DADA) pipeline to remove non-biological nucleotides^57^.

The output of the DADA2 pipeline resulted in a feature table of amplicon sequence variants (ASVs). This ASV table was then subjected to alpha and beta diversity analysis using the phyloseq and microbiomeSeq (http://www.github.com/umerijaz/microbiomeSeq) packages in R^58^. To assess differences in alpha-diversity measures among sample categories, variance analysis (ANOVA) was conducted using the plot_anova_diversity function in the microbiomeSeq package. For beta diversity analysis, Permutational Multivariate Analysis of Variance (PERMANOVA) with 999 permutations was performed on all principal coordinates derived from Canonical Correspondence Analysis (CCA) using the ordination function of the microbiomeSeq package. Additionally, correlation analysis between microbiome (genera) and metabolomics (metabolites) data was performed using the microbiome Seq package.

### Statistical analysis

Differential abundance analysis was conducted using the random-forest algorithm, which was implemented in the DAtest package (https://github.com/Russel88/DAtest/wiki/usage#typical-workflow). We compared differentially abundant methods using several metrics, including False Discovery Rate (FDR), Area Under the Curve (AUC) for Receiver Operating Characteristic (ROC) analysis, Empirical power (Power), and False Positive Rate (FPR).

Based on DAtest’s benchmarking results, we selected lefseq and Anova as our preferred methods for performing differential abundance analysis. Throughout the analysis, we considered statistical significance at *p* < 0.05 and adjusted *p* values for multiple comparisons using the Benjamini and Hochberg method to control the False Discovery Rate^59^.

For assessing associations between microbiome, and metabolites with metadata variables, we employed linear regression as a parametric test and the Wilcoxon test as a non-parametric test using the following cut-offs: alpha = 0.05, multiple correlation = false discovery rate or FDR, detection limit = 10^−10^. Potential confounding factors were assessed using PERMANOVA. These statistical analyses were carried out in R (version 4.1.2; R Core Team, 2021)^60^.

### Machine learning

Microbiome and untargeted metabolomics data from eoCRC vs aoCRC cohorts were used to construct a robust machine-learning classifier model for the groups using DIABLO (Data Integration Analysis for Biomarker Discovery using Latent variable approaches for Omics studies). DIABLO integrates molecular signatures such that the associations between metabolome and microbiome are sought in classifier optimization^42^. The number of components was selected using Mahalanobis distance. The final model was tuned to determine optimal parameters using leave-one-out cross-validation scores. Feature analysis was performed using the Block Rank algorithm. Features were ranked based on the stability metric, defined as a fraction indicating the number of models in the performance test that selected this feature when being fitted.

### Network analysis

To assess the biological relevance of metabolites, considering their microbial or dual (microbial or host-related) origin, we employed a differential microbe-metabolite interaction network. These networks were constructed using all microbial taxonomy and metabolomics features that were not preselected from the previously described model (DIABLO). Normalization was carried out through variance and stabilizing transformation. The SPRING method served as an association measure^13^. Estimated partial correlations were transformed into dissimilarities using the “signed” distance metric, and the resulting similarities were utilized as edge weights. Eigenvector centrality was employed to identify hubs and scale node sizes. Differential association analysis was conducted using Fisher’s *z*-test. Clusters were identified through greedy modularity optimization, with nodes unconnected in both groups subsequently removed.

### Supplementary information

Supplementary Information

## Data Availability

Authors T.J., N.S., A.A.K., and S.K. had full access to all the data in the study. We take full responsibility for the integrity of the data. There are restrictions to the availability of some data generated in this study due to the lack of authorization in our informed consent to share data beyond our institution without the explicit consent of the research subjects. The raw 16S rRNA amplicon sequencing data are available at: Sangwan and Khorana^61^. The data analyzed in this study are available from the corresponding author on reasonable request, S.D.K.
