# Supplementary material for: Multi-omics machine learning to study host-microbiome interactions in early-onset colorectal cancer
Source: NPJ Precis Oncol. 2024 Jul 17;8:146. doi: 10.1038/s41698-024-00647-1 (PMC11255257; doi:10.1038/s41698-024-00647-1)
Supplement: Supplementary file 1 — Supplementary Information [file 41698_2024_647_MOESM1_ESM.pdf]

**Supplementary Information**

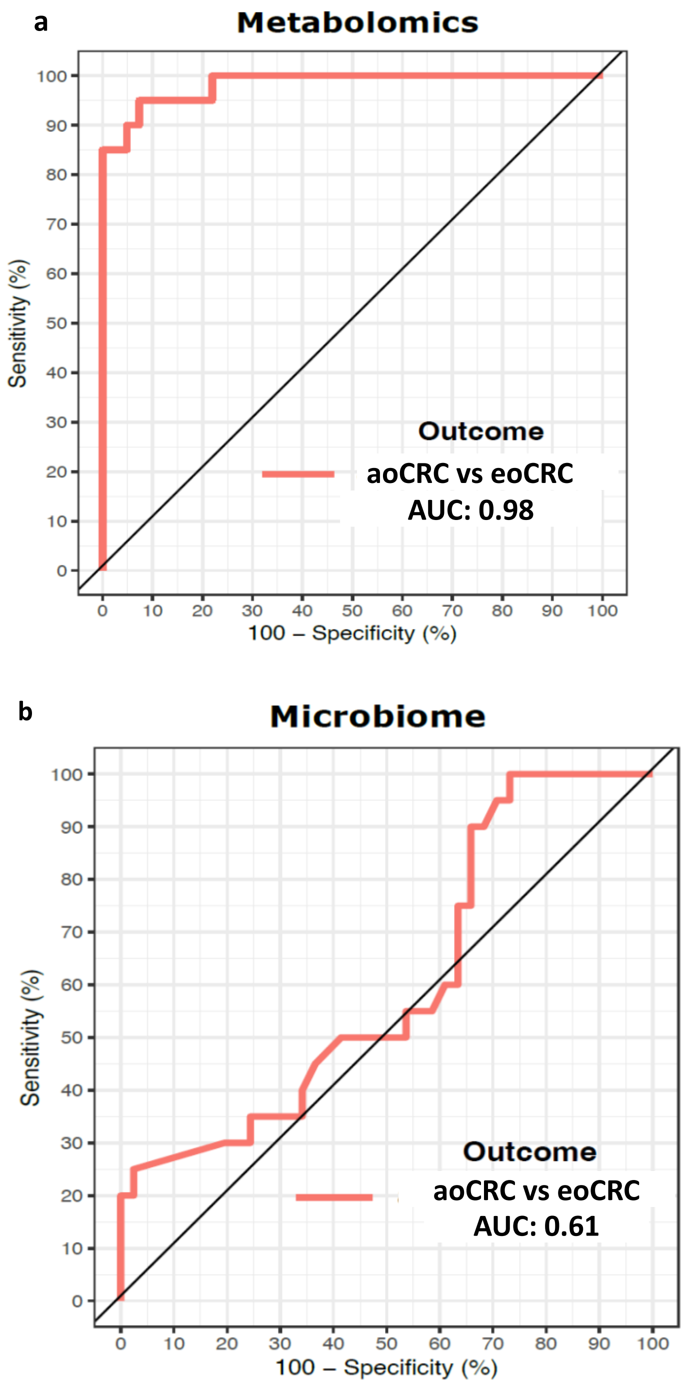


**Supplementary Figure 1.** AUC Plots Comparing The Classifier Performance of a. Metabolomics b. Microbiome

a

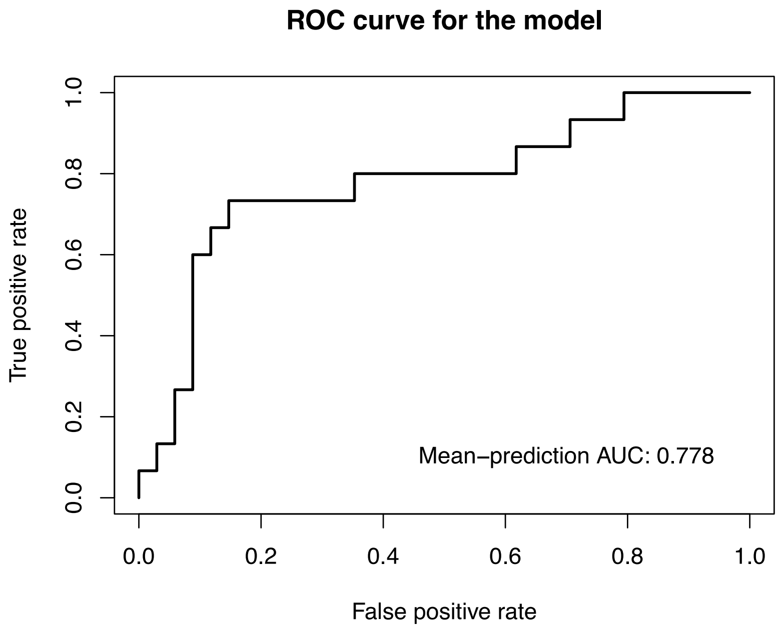


b

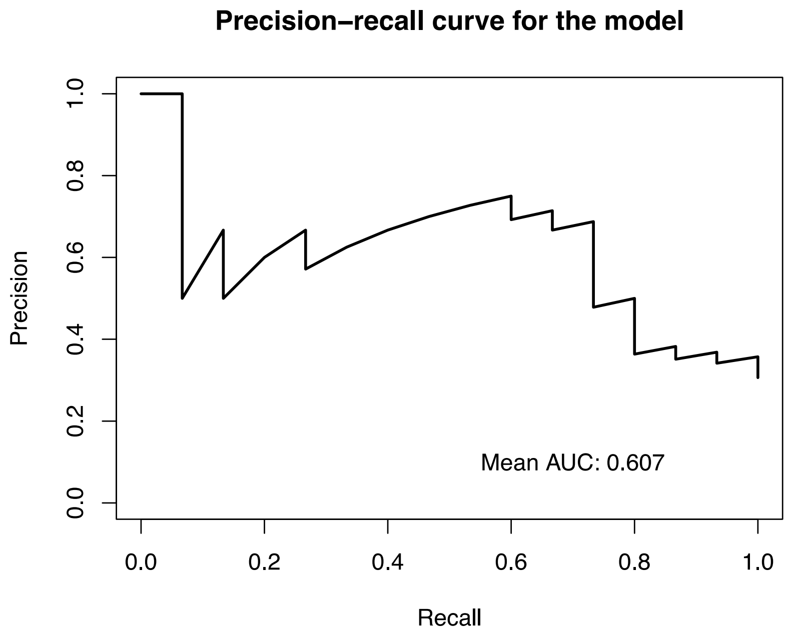


**Supplementary Figure 2.** Plots Comparing the Classifier Performance for Control Group (Individuals without CRC) a. AUC Plot b. Precision-Recall Curve
